# New Iridoid Derivatives from the Fruits of *Cornus officinalis* and Their Neuroprotective Activities

**DOI:** 10.3390/molecules24030625

**Published:** 2019-02-11

**Authors:** Lin-lin Ji, Xin Wang, Jin-jie Li, Xiang-jian Zhong, Bo Zhang, Jing Juan, Xiao-ya Shang

**Affiliations:** Beijing Key Laboratory of Bioactive Substances and Functional Foods, Beijing Union University, Beijing 100191, China; jll5927@163.com (L.-l.J.); shtwanxin@buu.edu.cn (X.W.); lijinjie.7785004@163.com (J.-j.L.); xiangjzhong@163.com (X.-j.Z.); zhangboiww@163.com (B.Z.); juanjingj2@163.com (J.J.)

**Keywords:** *Cornus officinalis*, iridoid, neuroprotective activity

## Abstract

Three previously undescribed iridoids, cornusfurals A–C, were isolated from the ethanolic extracts of fruits of *Cornus officinalis*. Their structures were elucidated by spectroscopic methods, including one-dimensional and two-dimensional nuclear magnetic resonance, ultraviolet spectroscopy, infrared spectroscopy, and mass spectrometry. The neuroprotective activity was evaluated by measuring corticosterone-induced damage in PC12 cells. The results showed that cornusfural B decreased corticosterone-induced PC12 cell damage compared with that in model cells.

## 1. Introduction

*Cornus officinalis* (Cornus) is a shrub distributed in Eastern Asia. The fruits of *C. officinalis*, named “shanzhuyu”, have traditionally been used for nourishing the liver and kidneys for thousands of years [1]. Extracts or constituents of *C. officinalis* have been reported to possess neuroprotective, antidiabetic, anti-inflammatory, antioxidant, and cardiovascular effects [2,3,4,5,6]. Phytochemical investigation of the fruits of *C. officinalis* has demonstrated the presence of iridoid glycosides, flavonoids, terpenoids, and polyphenols [7,8,9,10,11]. Among these, iridoids were found to be the most important constituents and are considered to be active components of the extracts. 

Depression is a common mental disorder characterized by persistent depression, which imposes a great burden on patients. Despite the high incidence of depression, the pathogenesis of this disease has not been fully elucidated [12]. The identification of antidepressant drugs from natural products is an important step in the development of novel therapeutics. PC12 cells, derived from the pheochromocytoma of the adrenal medulla in rats, are widely used in studies because of typical neuron characteristics [13]. Corticosterone-induced PC12 neuronal damage is useful as an in vitro experimental model for depression studies [14]. Loganin, the main iridoid glycoside from *C. officinalis*, has been reported to have antidepressive activity in recent studies [15,16]. Moreover, investigations of iridoid analogs from *C. officinalis* have revealed a number of biologically and structurally interesting compounds. In our previous pharmacology studies, we found that the macroporous resin 40% ethanol elution fraction of the ethanol extract of *C. officinalis* exhibited potent neuroprotective activity, and four new iridoid glycosides were isolated [17]. According to the HPLC spectroscopic characteristics, there are still many similar constituents which were suspected to have potential activities in this fraction. Thus, the 40% ethanol elution fraction was further evaluated in this study. Herein, the new iridoids were isolated, and their biological activities were discussed.

## 2. Results and Discussion

### 2.1. Characterization

**Figure 1:**
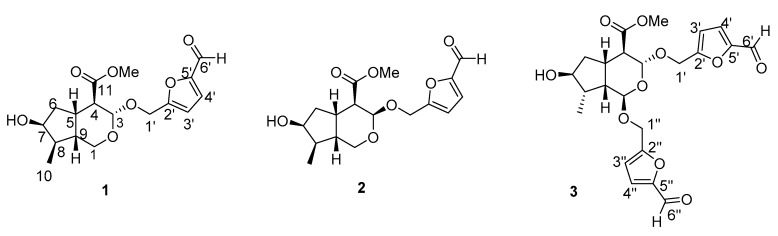
The structures of compounds **1**–**3**.

The crude extract of the fruits of *C. officinalis* was divided into five fractions by macroporous resin column chromatography. The generated 40% ethanol elution fraction was further isolated by the combination of silica gel column chromatography, low-pressure liquid chromatography, Sephadex LH-20 chromatography, and HPLC, generating three new compounds (Figure 1).

Cornusfural A (**1**) was obtained as an amorphous white solid. The molecular formula C_17_H_22_O_7_ was deduced from the quasimolecular ion peak at *m*/*z* 361.1247 [M + Na]^+^ (calcd 361.1257) in the high-resolution electrospray ionization mass spectrometry (HRESIMS) with an unsaturation of seven. The IR spectrum displayed the presence of hydroxyl (3257 cm^−1^) and carbonyl (1727, 1672 cm^−1^) groups. The ^1^H-NMR data of **1** (Table 1) indicated the presence of nine methine protons, including two oxygenated methines at δ_H_ 4.69 (1H, d, *J* = 8.4 Hz) and 4.09 (1H, m); two olefinic methines at δ_H_ 6.57 (1H, d, *J* = 3.5 Hz) and 7.34 (1H, d, *J* = 3.5 Hz); three aliphatic methines at δ_H_ 2.36 (1H, m), 1.80 (1H, m), and 1.84 (1H, m); one aldehyde at δ_H_ 9.52 (1H, s); three methylenes at δ_H_ 3.75 (1H, dd, *J* = 5.0, 12.1 Hz), 3.82 (1H, dd, *J* = 5.0, 12.1 Hz), 1.75 (1H, m), 1.79 (1H, m), 4.62 (1H, d, *J* = 13.6 Hz), and 4.71 (1H, d, *J* = 13.6 Hz); one methoxy at δ_H_ 3.61 (3H, s); and one methyl at δ_H_ 0.95 (3H, d, *J* = 6.6 Hz). ^13^C-NMR data gave 17 carbons, including one methyl (δ_C_ 12.4), two oxygenated methylenes (δ_C_ 64.9, 62.7), two oxygenated methines (δ_C_ 101.6, 75.5), two carbonyl carbons (δ_C_ 174.8, 179.5), and four olefinic carbons (δ_C_ 159.5, 154.2, 124.4, 112.7), as detailed in Table 1.

**Figure 2:**
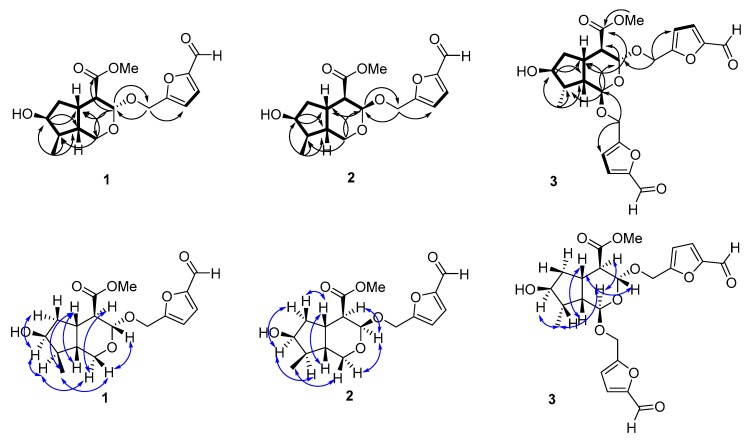
The HMBC (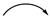), ^1^H-^1^H COSY (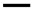) and NOESY (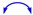) spectra of compounds.

In combination with analysis of the ^1^H-^1^H COSY spectrum (Figure 2), the NMR data showed that there was a four-spin system involving C3-C4-C5-C6-C7-C8-C9-C1, C5-C9, C8-C10, and C3′-C4′ in **1**. In the HMBC spectrum, the correlations of H-1/C-3, C5, and C-8; H-3/C-1, C-5 and C-11, H-7/C-5, and C-9; and H-10/C-7, C-8, and C-9 determined the cyclopentane iridoid carbon skeleton. In addition, a set of a 5-hydroxymethyl furfural moiety, which was proven by its characteristic signals at δ_H_ 4.62 (1H, d, *J* = 13.6 Hz), 4.71 (1H, d, *J* = 13.6 Hz), 6.57 (1H, d, *J* = 3.5 Hz), 7.34 (1H, d, *J* = 3.5 Hz), and 9.52 (1H, s), was present in **1**. The location of the 5-hydroxymethyl furfural group was established by the key cross-peaks from H-1′ to C-3 and from H-3 to C-1′ in the HMBC spectrum.

The relative configuration of **1** was deduced as depicted by the NOESY spectrum and coupling constants. The *J*_3,4_ = 8.4 Hz and *J*_4,5_ = 12.1 Hz suggested that the methoxycarbonyl group was β-equatorial [18,19], whereas the position of the furfural unit was α-equatorial. In the NOESY spectrum, correlations of H-3/H-1β/H-10/H-5/H-9 and H-4/H-1α/H-8/H-7/H-6α indicated that H-3, H-5, and H-9 were of the β-configuration, whereas the H-4, H-7, and H-8 were of the α-configuration. The absolute configuration of **1** was substantiated based on biogenetic grounds in that nearly all iridoids found in nature have a configuration of 5*S* and 9*R*, and by analogy to the known compounds that were found to have closely comparable NMR data and similar optical rotation values [18]. The ECD spectrum of **1** exhibited negative Cotton effects at 234 and 272 nm arising from 5-hydroxymethylfurfural (Supplementary Materials). Thus, the configurations of H-3/4/5/7/8/9 should be 3*S*/4*R*/5*S*/7*S*/8*R*/9*R*. Therefore, the structure of **1** was established as cornusfural A.

Compound **2** was found to have the same formula (C_17_H_22_O_7_) as **1** with the HRESIMS ion at *m*/*z* 361.1247 [M + Na]^+^ (calcd for 361.1257, C_17_H_22_O_7_Na) and required seven degrees of unsaturation. Moreover, this compound exhibited similar IR, UV, and NMR data as **1**, suggesting their structural resemblance. However, the upfield shifts of C-1 (δ_C_ 58.9), C-3 (δ_C_ 97.3), C-4 (δ_C_ 49.2), and C-5 (δ_C_ 32.5) and the coupling constants *J*_3,4_ = 3.8 Hz and *J*_4,5_ = 11.9 Hz showed that the iridoid skeleton of **2** was an epimer of **1**, which was confirmed by the NOESY correlations of H-3 with H-4 and H-1α. Thus, the spectroscopic data of **2** indicated that this compound was an epimer of **1** and that the configuration of H-3 was α-oriented. Compared to compound **1**, the absolute configuration of **2** was (3*R*, 4*R*, 5*S*, 7*S*, 8*R,* and 9*R*). Hence, the structure of **2** (cornusfural B) was established as shown.

Compound **3** was obtained as a white powder. The molecular weight was determined by HRESIMS, which showed an [M + Na]^+^ ion at *m*/*z* 485.1407 (calcd for 485.1418, C_32_H_38_O_15_Na), indicating 11 degrees of unsaturation. The IR spectrum showed absorption bands due to hydroxy (3256 cm^−1^) and carbonyl (1727 and 1669 cm^−1^) groups. The ^1^H-NMR spectrum of compound **3** showed that there was a doublet methyl group δ_H_ 0.97 (3H, d, *J* = 6.3 Hz); three pairs of geminal coupled methylene groups δ_H_ 1.67–1.72 (1H, m, H-6α), 1.80–1.82 (1H, m, H-6β), 4.70 (1H, d, *J* = 13.3Hz, H-1′α), 4.78 (1H, d, *J* = 13.3Hz, H-1′β), 4.69 (1H, d, J = 13.4 Hz, H-1′′α), and 4.81 (1H, d, *J* = 13.4 Hz, H-1′′β); four methine protons δ_H_ 2.30 (1H, dd, *J* = 8.6, 12.3 Hz, H-4), 2.49–2.53 (1H, m, H-5), 1.84 (1H, m, H-8), and 1.88 (1H, o, H-9); an oxygenated methine proton δ_H_ 4.08–4.09 (1H, m, H-7); two acetal protons δ_H_ 4.95 (1H, d, *J* = 2.9 Hz, H-1) and 5.07 (1H, d, *J* = 8.6 Hz, H-3); four olefinic protons δ_H_ 6.67 (1H, d, *J* = 3.6 Hz, H-3′), 7.36 (1H, d, *J* = 3.6 Hz, H-4′), 6.63 (1H, d, *J* = 3.6 Hz, H-3′′), and 7.35 (1H, d, *J* = 3.6 Hz, H-4′′); and two aldehyde groups δ_H_ 9.54 (1H, s, H-6′) and 9.52 (1H, s, H-6′′). There were 23 carbons found in the ^13^C-NMR spectrum, and there were three carbonyl groups, that is, δ_C_ 174.4 (C-11), 179.5 (C-6′), and 179.5 (C-6′′). The ^1^H-^1^H COSY spectra found correlations of H-3/H-4/H-5, H-5/H-6/H-7/H-8, H-8/H-9/H-10, and H-5/H-9. In the HMBC spectrum, the correlations of H-1 to C-3/C-5/C-8, H-3 to C-5/C-11, H-7 to C-5/C-6/C-8/C-9, and H-10 to C-7/C-8/C-9 indicated that compound **3** was an iridoid-type compound. According to the spectra and correlations, there were two additional 5-hydroxymethylfurfural groups connecting to the iridoid skeleton. The HMBC correlations from H-1′ to C-3 and from H-1′′ to C-1 revealed that the 5-hydroxymethylfurfural moieties were present at C-1 and C-3, respectively. 

The relative stereochemistry of **3** was determined by the NOESY spectrum and coupling constants. The large coupling constant *J*_4,5_ = 11.4 Hz between H-4 and H-5 indicated that H-4 was located in the α-oriented configuration, and *J*_3,4_ = 8.6 Hz indicated that H-3 was β-oriented. In the NOESY spectrum, correlations between H-3 and H-5/H-9 indicated that H-5 and H-9 were both β-oriented. Moreover, NOESY correlations of H-4 with H-1 and of H-10 with H-1 and H-7 confirmed that H-1, H-10, and H-7 were all α-oriented. According to the biogenetic grounds in which the absolute configurations of H-5 and H-9 were 5*S* and 9*R*, respectively, and because the negative Cotton effects were at 234 and 281 nm, it can be said the compound **3** had an absolute configuration of (1*R*, 3*R*, 4*R*, 5*S*, 7*S*, 8*S,* and 9*R*). Analysis of the HSQC, ^1^H-^1^H COSY, and HMBC spectra led to the complete assignments of the proton and carbon signals in compound **3**. Therefore, compound **3** was characterized as cornusfural C.

### 2.2. Neuroprotective Effects of Compounds ***1**–**3***

Corticosterone-induced PC12 neuronal damage is useful as an in vitro experimental model for depression studies [14]. The neuroprotective effects of compounds **1**–**3** were assessed (Table 2). Compound **2** exhibited neuroprotective activity compared with the model (complete medium with 500 μM corticosterone).

### 2.3. Discussion

As the results showed, only compound **2** exhibited neuroprotective activity in in vitro experiments. The structural analysis of these new compounds showed that compounds **1** and **3** had the same configuration of 3α, while compound **2** had a 3β configuration. The current data showed that the neuroprotective activity of these iridoid derivatives might be associated with the stereochemistry of C-3, and the 3β-substituents might be the active groups. However, the mechanism of the effect of C-3 configuration needs further confirmation by studying the structure–activity relationships of more similar compounds. Although there are many reports on the neuroprotective activities of iridoids from *C. officinalis*, the structure–activity relationship has not been reported [20,21,22]. Morroniside, the main active component of *C. officinalis*, was even evaluated in the form of a mixture in the neuroprotective study. Therefore, more efforts are suggested to explore the structure–activity relationships of iridoids from *C. officinalis.*

## 3. Materials and Methods

### 3.1. Plant Material

The fruits of *C. officinalis* were purchased from Tong-Ren-Tang Company in Beijing, People’s Republic of China, and were authenticated by Professor Wen Wang, Xuanwu Hospital of Capital Medical University. Voucher specimen number 20090305 was deposited at the Beijing Union University, Beijing Key Laboratory of Bioactive Substances, and Functional Foods, Beijing, China.

### 3.2. General Experimental Procedures

The HRESIMS data were generated on a Thermo QE Spectrometer (Thermo Scientific Inc., Waltham, MA, USA). The specific rotation data were obtained with a JASCO P-2000 polarimeter (JASCO Inc., Tokyo, Japan). IR spectra were recorded as KBr disks on a Nicolet Impact 400 FT-IR Spectrophotometer (Nicolet Instrument Inc., Madison, WI, USA). The circular dichroism spectra and UV data were recorded on a JASCO J-1005 circular dichroism spectrometer (JASCO Corporation, Tokyo, Japan). The one- and two-dimensional NMR spectra were recorded in CD_3_OD with TMS as the internal standard on Varian 500 MHz and Bruker AV500-III spectrometers (Bruker Corporation, Billerica, MA, USA). A Waters 2996 series was coupled with an RP-C18 column (Sunfire, 250 mm × 19 mm i.d.; Alltech Associates, Inc., Bannockburn, IL, USA) and a Waters 2998 dual-wavelength absorbance detector (Waters Corporation, Milford, MA, USA). Column chromatography was performed with silica gel (160–200 mesh; Qingdao Marin Chemical Inc., Qingdao, China) and Sephadex LH-20 (Pharmacia Biotech AB, Uppsala, Sweden). Thin-layer chromatography (TLC) was used with glass precoated silica gel GF254 plates. Spots were visualized under UV light or by spraying with 8% H_2_SO_4_ in 95% EtOH followed by heating.

### 3.3. Cell lines, Chemicals, and Biochemicals

PC12 cells (adrenal gland; pheochromocytoma) were purchased from the Shanghai Institute of Biochemistry and Cell Biology, CAS (Shanghai, China). DMSO, corticosterone, and MTT were obtained from Sigma (St. Louis, MO, USA). The methanol used for HPLC isolation, which was of HPLC grade, was purchased from Fisher (Waltham, MA, USA). The solvents used to open the column isolation (Silica gel and Sephadex LH-20 gel column) in the study, such as chloroform, acetone, and methanol, were of ACS grade (Beijing, China). 

### 3.4. Extraction and Isolation

Air-dried fruits (10 kg) of *C. officinalis* were exhaustively extracted with 50% aqueous solution (100 L × 3, 1 h) at reflux. The ethanolic extracts were concentrated under reduced pressure to dryness. The residue was suspended in H_2_O and applied to a Diaion HP-20 column chromatography (Mitsubishi Chemical Corporation, Nagasaki, Japan) by a stepwise gradient of EtOH/H_2_O (0:100, 20:80, 40:60, 70:30, and 95:5, *v*/*v*) to yield five fractions (fractions A–E). The separation of fraction B (EtOH:H_2_O = 40:60, 500 g) was carried out on a silica gel column eluted with CHCl_3_/MeOH (15:1 to 8:1, *v*/*v*) to afford four major fractions (B1–B4) based on TLC analysis. The separation of fraction B1 (73 g) was carried out on a silica gel column eluted with CHCl_3_/MeOH (50:1 to 3:1) to provide four subfractions B1-1–B1-4. Fraction B1-2 (42.2 g) was subjected to silica gel column chromatography, eluted with CHCl_3_/MeCOMe (10:1 to 2:1, *v*/*v*), to give three subfractions B1-2-1–B1-2-3. Fraction B1-2-1 (10.1 g) was separated using a Sephadex LH-20 column eluted with CHCl_3_/MeOH (2:1) as the mobile phase to give three subfractions B1-2-1-1–B1-2-1-3. Fraction B1-2-1-2 (3.4 g) was further fractionated over a Sephadex LH-20 column eluted with petroleum ether/CHCl_3_/MeOH (5:5:1) as the mobile phase, and the subfractions were purified by preparative HPLC using 60% MeOH/H_2_O (18 mL/min) to yield compounds **1** (18 mg) and **2** (11 mg). Fraction B1-2-3 was subjected to CombiFlash HCN silica gel column chromatography by a gradient elution with petroleum ether/acetone (20:1, 10:1, 8:1, 6:1, 3:1, 1:1) to yield five fractions B1-2-3-1–B1-2-3-5. Fraction B-1-2-3-3 was purified by preparative HPLC using 55% MeOH/H_2_O (18 mL/min) as the mobile phase to yield compound **3** (9 mg).

### 3.5. Compounds Characterization Data

Cornusfural A (**1**): White amorphous powder, [α${]}_{D}^{25}$ −121.2 (*c* 0.06, CH_3_OH); UV (MeOH) λ_max_ (log ε): 230 (4.01) nm, 280 (4.21) nm; CD (MeOH) 234 (Δε −1.49), 272 (Δε −6.31) nm; IR ν_max_ 3257, 2959, 2872, 1727, 1672, 1521, 1457 cm^−1^; ^1^H-NMR (methanol-*d*_4_, 500 MHz) and ^13^C-NMR (methanol-*d*_4_, 125 MHz) spectral data see Table 1; (+)-HRESIMS *m*/*z* 361.1247 [M + Na]^+^ calcd for 361.1257 C_17_H_22_O_7_ Na). 

Cornusfural B (**2**): White amorphous powder, [α${]}_{D}^{25}$ +120 (*c* 0.06, CH_3_OH); UV (MeOH) λ_max_ (log ε): 230 (3.85) nm, 280 (4.11) nm; CD (MeOH) 234 (Δε +4.11), 281 (Δε +13.5) nm; IR ν_max_ 3431, 2954, 2877, 1736, 1675, 1521, 1436 cm^−1^; ^1^H-NMR (methanol-*d*_4_, 500 MHz) and ^13^C-NMR (methanol-*d*_4_, 125 MHz) spectral data see Table 1; (+)-HRESIMS *m*/*z* 361.1247 [M + Na]^+^ (calcd for 361.1257, C_17_H_22_O_7_Na).

Cornusfural C (**3**): White amorphous powder, [α${]}_{D}^{25}$ −44.2 (*c* 0.03, CH_3_OH); UV (MeOH) λ_max_ (log ε): 232 (3.80) nm, 280 (4.02) nm; CD (MeOH) 234 (Δε −3.11), 281 (Δε −13.9) nm; IR ν_max_ 3256, 2959, 2926, 1727, 1669, 1523, 1458 cm^−1^; ^1^H-NMR (methanol-*d*_4_, 500 MHz) and ^13^C-NMR (methanol-*d*_4_, 125 MHz) spectral data see Table 1; (+)-HRESIMS *m*/*z* 485.1407 [M + Na]^+^ (calcd for 485.1418, C_32_H_38_O_15_Na).

### 3.6. Neuroprotection Bioassays

PC12 cells were cultured in RPMI 1640 medium supplemented with 1% streptomycin, 5% horse serum, and 5% fetal bovine serum. The cell suspensions were seeded in 96-well culture plates (2 × 10^4^ cells/mL) and cultured for 24 h. Then, the medium was replaced with different fresh media, including the control (complete medium), the model (complete medium with 500 μM corticosterone), and the sample (the test compounds at a concentration of 10 μM), and the cells were cultured for 24 h. Next, 10 μL MTT (5 mg/mL) was added to each well. After incubation for 2 h, the medium was removed, and 100 μL DMSO was added to dissolve the formazan crystals generated by the reaction. The optical density was then measured on a microplate reader (Molecular Devices, SFO, USA) at 570 nm. Cell viability was indicated as a percentage of the control.

## 4. Conclusions

Three new iridoids, that is, cornusfural A (**1**), cornusfural B (**2**), and cornusfural C (**3**), containing a furan ring, were isolated from the fruits of *C. officinalis*, and the neuroprotective effects of these compounds were evaluated. Compound **2** showed neuroprotective activities. The neuroprotective activities of iridoid glycosides have been evaluated through a variety of in vitro and in vivo studies [20,21,22]; however, iridoid aglycones isolated from *C. officinalis* have rarely been studied. Importantly, we identified one new iridoid aglycone exhibiting neuroprotective effects, thereby providing a potential new neuroprotective agent for further antidepressants research. According to the literature, iridoid glycosides are the main component in this plant, but the quality of iridoid aglycones are less. If the iridoid aglycones have significant biological activities, a large number of aglycones can be obtained through the hydrolysis of iridoid glycosides, which could be used for further animal experiments. This indicated that *Cornus officinalis* is a good resource of bioactive compounds and functional food.

## Figures and Tables

**Table 1:** ^1^H-NMR and ^13^C-NMR spectroscopic data of compounds **1**–**3**^a^.

No.	Compound 1		Compound 2		Compound 3	
	δ_H_ (*J* in Hz)	δ_C_	δ_H_ (*J* in Hz)	δ_C_	δ_H_ (*J* in Hz)	δ_C_
1α	3.75 (dd, *J* = 5.0, 12.1)	64.9	3.99 (dd, *J* = 3.9, 12.0)	58.9	4.95 (d, *J* = 2.9)	101.3
1β	3.82 (dd, *J* = 5.0, 12.1)		3.54 (dd, *J* = 1.4, 12.0)			
3	4.69 (d, *J* = 8.4)	101.6	5.04 (d, *J* = 3.8)	97.3	5.07 (d, *J* = 8.6)	97.7
4	2.26 (dd, *J* = 8.4, 12.1)	52.5	2.48 (dd, *J* = 3.8, 11.9)	49.2	2.30 (dd, *J* = 8.6, 12.3)	51.7
5	2.36 (m)	39.1	2.65 (m)	32.5	2.51 (m)	37.4
6α	1.79 (m)	40.4	1.83 (m)	42.5	1.69 (m)	40.2
6β	1.75(m)		1.77 (m)		1.81 (m)	
7	4.09 (m)	75.5	4.08 (m)	74.6	4.08 (m)	74.9
8	1.80 (m)	40.7	1.90 (m)	39.7	1.84 (m)	40.9
9	1.84 (m)	43.5	1.68 (m)	42.9	1.88 (m)	47.8
10	0.95 (d, *J* = 6.6)	12.4	0.97 (d, *J* = 6.8)	12.2	0.97 (d, *J* = 6.3)	12.7
11	-	174.8	-	173.0	-	174.4
12	3.61 (s)	52.3	3.56 (s)	52.2	3.62 (s)	52.5
1′α	4.62 (d, *J* = 13.6)	62.7	4.53 (d, *J* = 13.7)	61.9	4.70 (d, *J* = 13.3)	63.1
1′β	4.71 (d, *J* = 13.6)		4.64 (d, *J* = 13.7)		4.78 (d, *J* = 13.3)	
2′	-	159.5	-	159.3	-	159.5
3′	6.57 (d, *J* = 3.5)	112.7	6.58 (d, *J* = 3.6)	113.1	6.67 (d, *J* = 3.6)	113.0
4′	7.34 (d, *J* = 3.5)	124.4	7.35 (d, *J* = 3.6)	124.4	7.36 (d, *J* = 3.6)	124.4
5′	-	154.2	-	154.3	-	154.3
6′	9.52 (s)	179.5	9.53 (s)	179.4	9.54 (s)	179.5
1″α					4.69 (d, *J* = 13.4)	62.8
1″β					4.81 (d, *J* = 13.4)	
2″					-	159.2
3″					6.63 (d, *J* = 3.6)	112.9
4″					7.35 (d, *J* = 3.6)	124.4
5″					-	154.3
6″					9.52 (s)	179.5

^a 1^H-NMR data (δ) were measured in methanol-*d*_4_ at 500 MHz and ^13^C-NMR data (δ) were measured in methanol-*d*_4_ at 125 MHz for compounds **1**-**3**. Coupling constants (*J*) in Hz are given in parentheses. The assignments were based on ^1^H-^1^H COSY, HSQC, HMBC and NOESY experiments.

**Table 2:** Neuroprotective effects of compounds **1**–**3** at a concentration of 10^−5^ M (means ± SD, *n* = 6).

Sample	Viability (%)
Control	100.00 ± 1.21
Model	53.54 ± 1.82 ^###^
**1**	57.42 ± 2.74
**2**	68.23 ± 2.26 ***
**3**	59.46 ± 3.62

^###^*p* < 0.01 vs. control, *** *p* < 0.05 vs. model.

## Supplementary Materials

The supplementary materials are available online.

## References

[1] Chinese Pharmacopoeia Commission (2015). Pharmacopoeia of the People’s Republic of China.

[2] Wang W., Xu J.D., Li L., Wang P.C., Ji X.M., Ai H.X., Zhang L. (2010). Neuroprotective effect of morroniside on focal cerebral ischemia in rats. Brain Res. Bull..

[3] Kang D.G., Choi D.H., Lee J.K., Lee Y.J., Moon M.K., Yang S.N., Kwon T.O., Kwon J.W., Kim J.S., Lee H.S. (2007). Endothelial NO/cGMP-dependent vascular relaxation of cornuside isolated from the fruits of *Cornus officinalis*. Planta Med..

[4] He K., Song S.H., Zou Z.Y., Feng D., Wang Y.Z., Li X.G., Ye X.L. (2016). The hypoglycemic and synergistic effect of loganin, morroniside, and ursolic acid isolated from the fruits of *Cornus officinalis*. Phytother. Res..

[5] An Y.A., Hwang J.Y., Lee J.S., Kim Y.C. (2015). Cornus officinalis methanol extract upregulates melanogenesis in melan-a cells. Eur. Toxicol. Res..

[6] Huwang K.A., Huang Y.J., Song J. (2016). Antioxidant activities and oxidative stress inhibitory effects of ethanol extracts from *Cornus officnalis* on raw 264.7 cells. BMC Complementary Altern. Med..

[7] Ma W., Wang K.J., Cheng C.S., Yan G.Q., Lu W.L., Ge J.F. (2014). Bioactive compounds from *Cornus officinalis* fruits and their effects on diabetic nephropathy. J. Ethnopharmacol..

[8] Ye X.S., He J., Cheng Y.C., Zhang L., Qiao H.Y., Pan X.G., Zhang J., Liu S.N., Zhang W.K., Xu J.K. (2017). Cornusides A-O, Bioactive iridoid glucoside dimers from the fruits of *Cornus officinalis*. J. Nat. Prod..

[9] Lee S.H., Tanaka T., Nonaka G.I., Nishioka I. (1989). Sedoheptulose digallate from *Cornus officnalis*. Phytochemistry.

[10] Lee J., Jang D.S., Kim N.H., Lee Y.M., Kim J., Kim J.S. (2011). Galloyl glucoses from the seeds of *Cornus officinalis* with inbibitory activity against protein glycation, aldose reductase, and cataractogenesis ex vivo. Biol. Pharm. Bull..

[11] Xie X.Y., Wang R., Shi Y.P. (2012). Chemical constituents from the fruits of *Cornus officinalis*. Biochem. Syst. Ecol..

[12] Ledford H.D. (2014). If depression were cancer. Nature.

[13] Mao Q.Q., Xian Y.F., Ip S.P., Tsai S.H., Che C.T. (2011). Protective effects of peony glycosides against corticosterone-induced cell death in PC12 cells through antioxidant action. Cell. Mol. Neurobiol..

[14] Li Y.F., Gong Z.H., Cao J.B., Wang H.L., Luo Z.P., Li J. (2003). Antidepressant-like effect of agmatine and its possible mechanism. Eur. J. Pharm..

[15] Zhang W.K., Xu J.K., He J., Qiao H.Y., Ye X.S. (2016). Application of Loganin in Manufacture of Medicine or Health Product for Preventing and Treating Depression, Anxiety and Other Mental Disorder Diseases. CN Patent.

[16] Rajabi M., Mohaddes G., Farajdokht F., Nayebi Rad S., Mesgari M., Babri S. (2018). Impact of loganin on pro-inflammatory cytokines and depression-and anxiety-like bhaviors in male diabetic rats. Physiol. Int..

[17] Wang X., Liu C.H., Li J.J., Zhang B., Ji L.L., Shang X.Y. (2018). Iridoid glycosides from the fruits of *Cornus officinalis*. J. Asian Nat. Prod. Res..

[18] Hu J., Mao X., Shi X.D., Li H. (2017). Chemical constituents of the barks of *Litsea rubescens*. Chem. Nat. Comp..

[19] Kocsis A., Szabo L.F. (1993). New bis-iridoids from *Dipsacs laciniatus*. J. Nat. Prod..

[20] Jeong E.J., Kim T.B., Yang H.J., Kang S.Y., Kim S.Y., Sung S.H., Kim Y.C. (2012). Neuroprotective iridoid glycosides from *Cornus officinalis* fruits against glutamate-induced toxicity in HT22 hippocampal cells. Phytomedicine.

[21] Zhao L.H., Ding Y.X., Zhang L., Li L. (2010). Cornel iridoid glycoside improve memory and promotes neuronal survival in fimbria-fornix transected rats. Eur. J. Pharm..

[22] Wang W., Sun F.L., An Y., Ai H.X., Zhang L., Huang W.T., Li L. (2009). Morroniside protects human neuroblastoma SH-SY5Y cells against hydrogen peroxide-induced cytotoxicity. Eur. J. Pharm..

