# Supplementary material for: New Iridoid Derivatives from the Fruits of Cornus officinalis and Their Neuroprotective Activities
Source: Molecules. 2019 Feb 11;24(3):625. doi: 10.3390/molecules24030625 (PMC6384786; doi:10.3390/molecules24030625)
Supplement: Supplementary file 1 [file molecules-24-00625-s001.pdf]

Supplementary Matrials

New Iridoid Derivatives from the Fruits of *Cornus officinalis* and Their Neuroprotective Activities

Lin-lin Ji[1#], Xin Wang[1#], Jin-Jie Li[1], Xiang-Jian Zhong[1], Bo Zhang[1], Jing Juan[1], Xiao-Ya Shang[1*]

[1] Beijing Key Laboratory of Bioactive Substances and Functional Foods, Beijing Union University, Beijing 100191, China

* Correspondence: shangxiaoya@buu.edu.cn; Tel.: +86-010-6200-4533

\# These authors contributed equally to this work

Received: date; Accepted: date; Published: date

Content List

		Pages
Compound **1**	^{1}H NMR spectrum of Compound **1**	S1
	^{13}C NMR spectrum of Compound **1**	S2
	HSQC spectrum of Compound **1**	S3
	^{1}H-^{1}H COSY spectrum of Compound **1**	S4
	HMBC spectrum of Compound **1**	S5
	NOESY spectrum of Compound **1**	S6
	HRESIMS spectrum of Compound **1**	S7
	IR spectrum of Compound **1**	S8
	UV spectrum of Compound **1**	S9
	CD spectrum of Compound **1**	S10
Compound **2**	^{1}H NMR spectrum of Compound **2**	S11
	^{13}C NMR spectrum of Compound **2**	S12
	HSQC spectrum of Compound **2**	S13
	^{1}H-^{1}H COSY spectrum of Compound **2**	S14
	HMBC spectrum of Compound **2**	S15
	NOESY spectrum of Compound **2**	S16

	HRESIMS spectrum of Compound **2**	S17
	IR spectrum of Compound **2**	S18
	UV spectrum of Compound **2**	S19
	CD spectrum of Compound **2**	S20
Compound **3**	^{1}H NMR spectrum of Compound **3**	S21
	^{13}C NMR spectrum of Compound **3**	S22
	HSQC spectrum of Compound **3**	S23
	^{1}H-^{1}H COSY spectrum of Compound **3**	S24
	HMBC spectrum of Compound **3**	S25
	NOESY spectrum of Compound **3**	S26
	HRESIMS spectrum of Compound **3**	S27
	IR spectrum of Compound **3**	S28
	UV spectrum of Compound **3**	S29
	CD spectrum of Compound **3**	S30

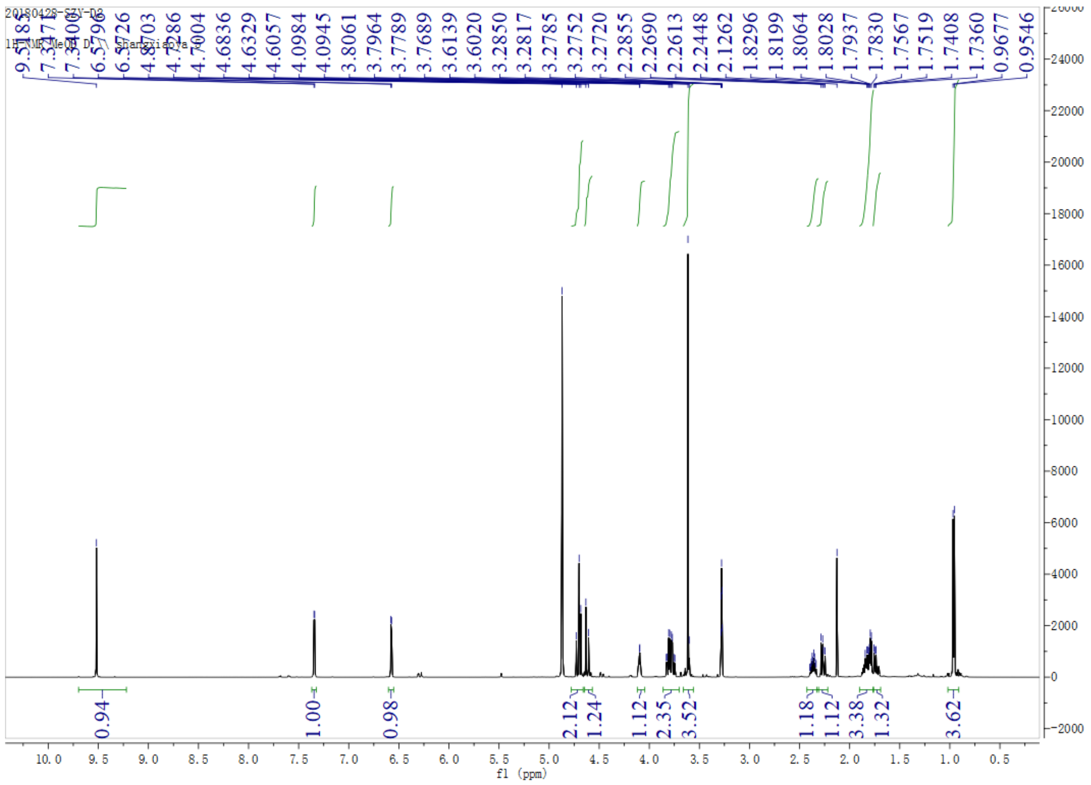

S1 ^{1}H NMR spectrum of Compound **1**

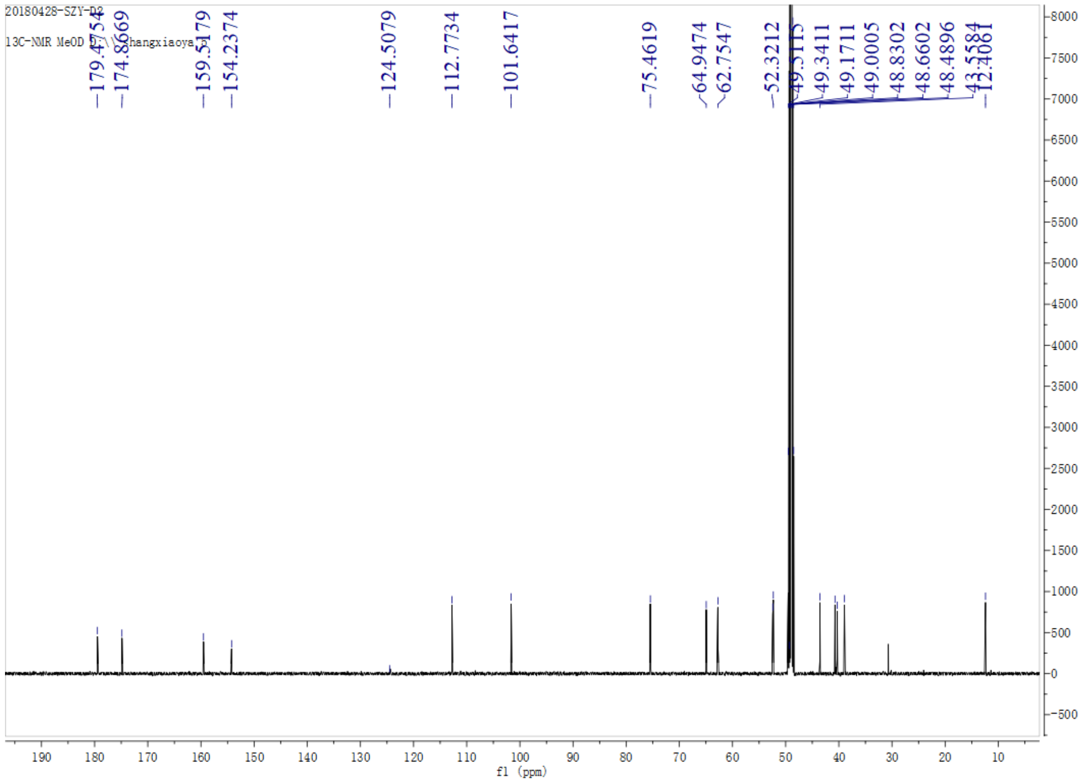

S2 ^{13}C NMR spectrum of Compound **1**

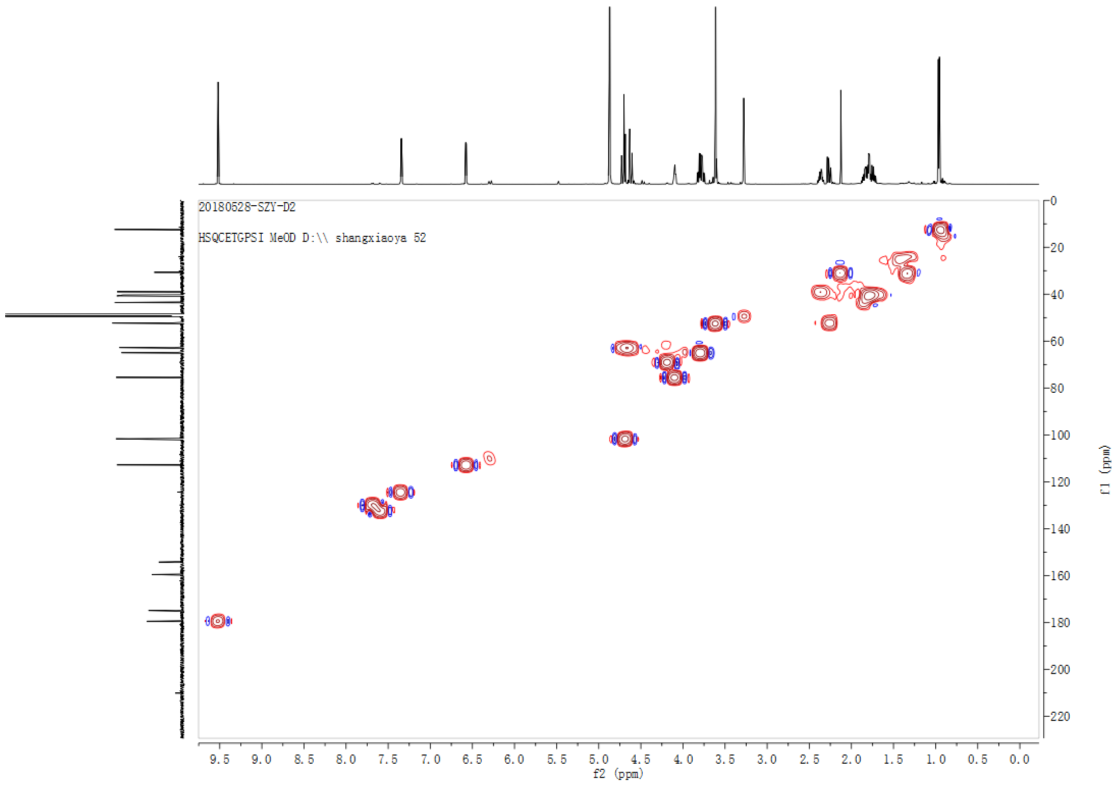

S3 HSQC spectrum of Compound **1**

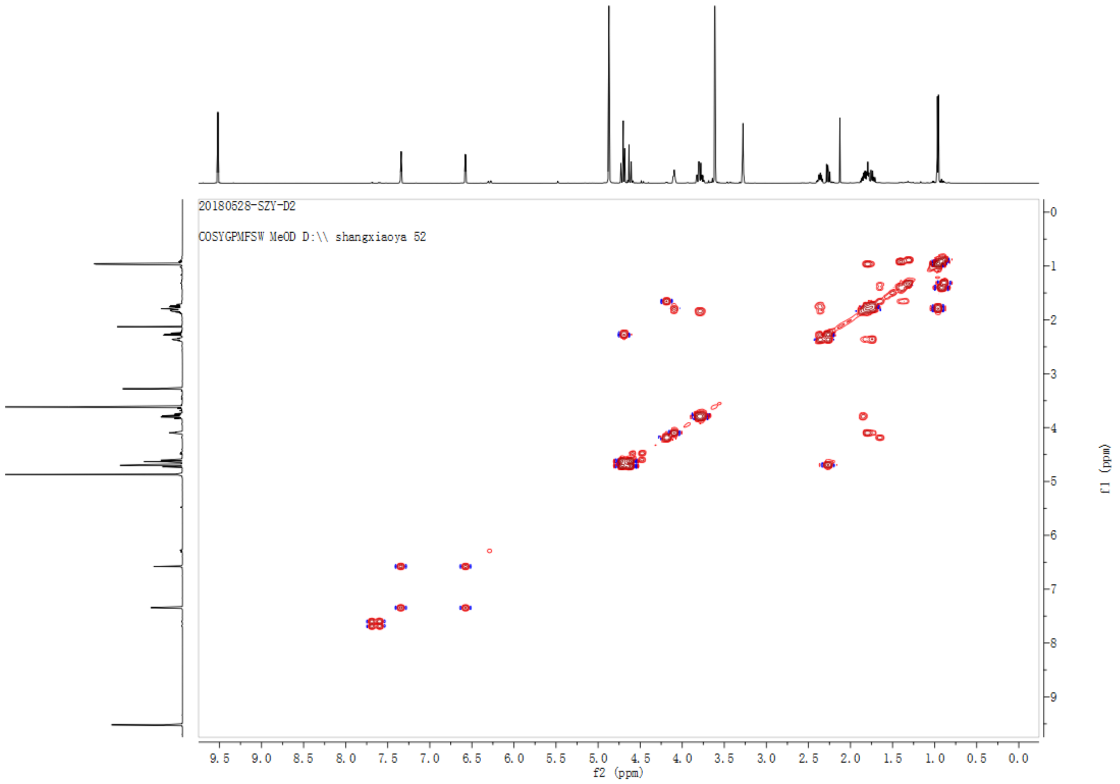

S4 ^{1}H-^{1}H COSY spectrum of Compound **1**

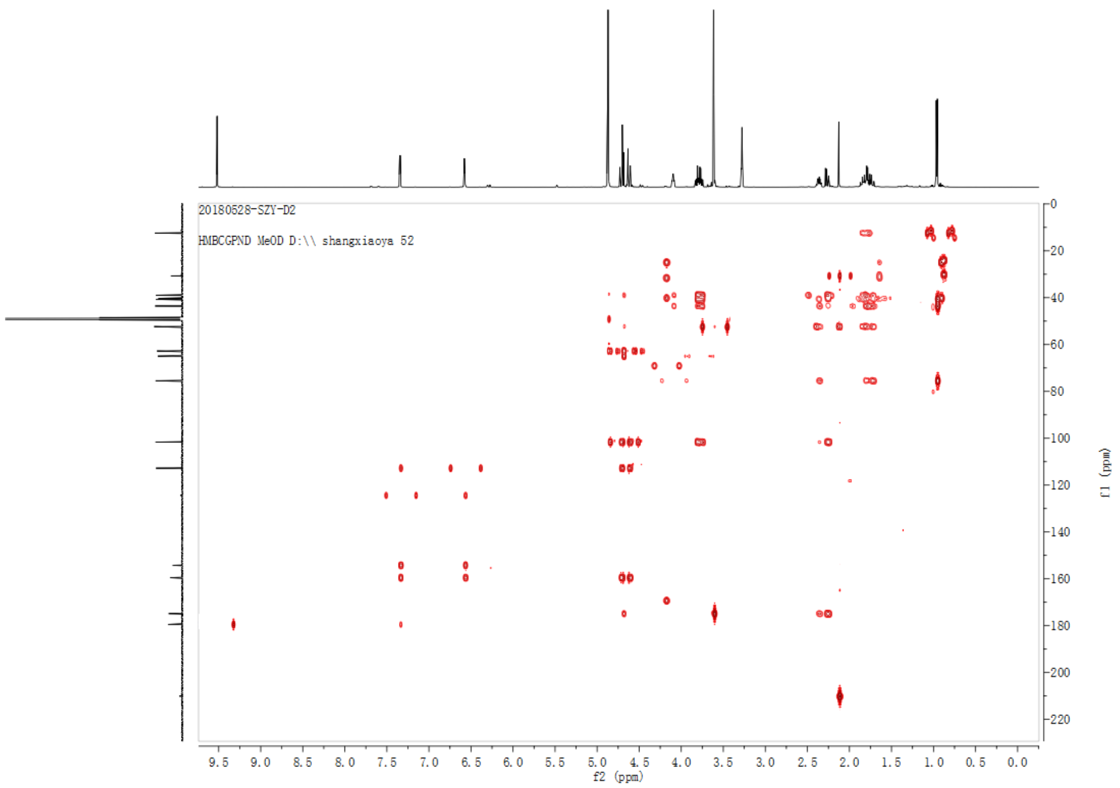

S5 HMBC spectrum of Compound **1**

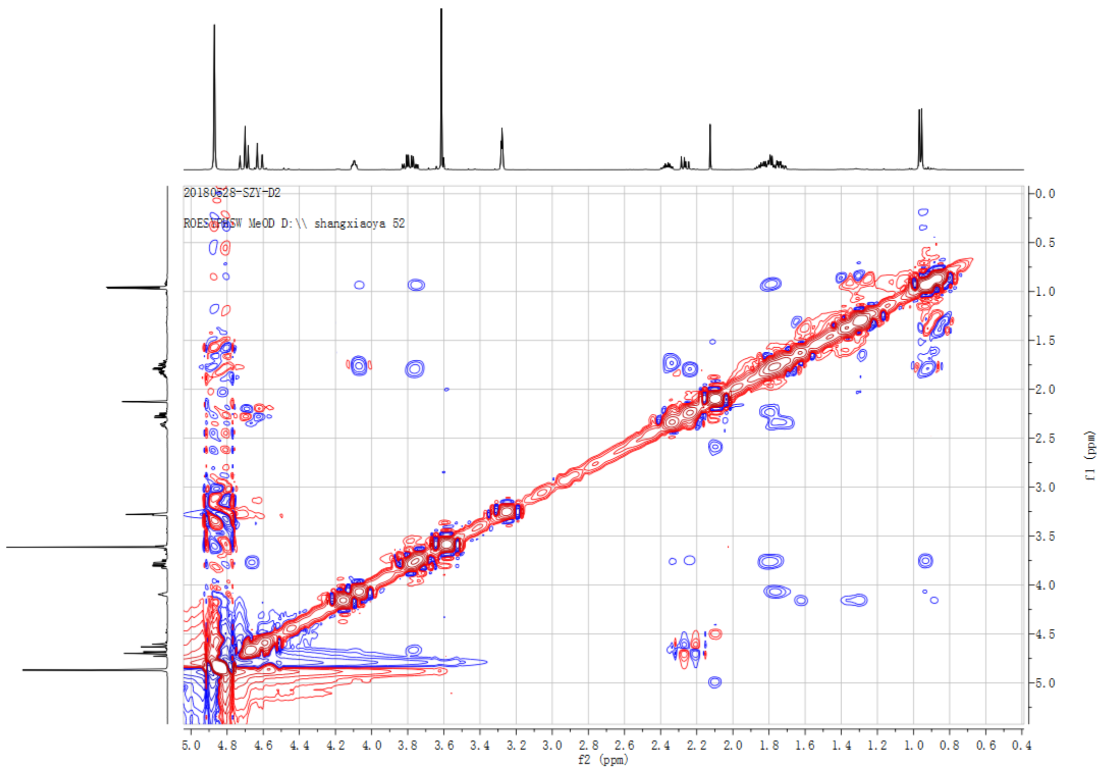

S6 NOESY spectrum of Compound **1**

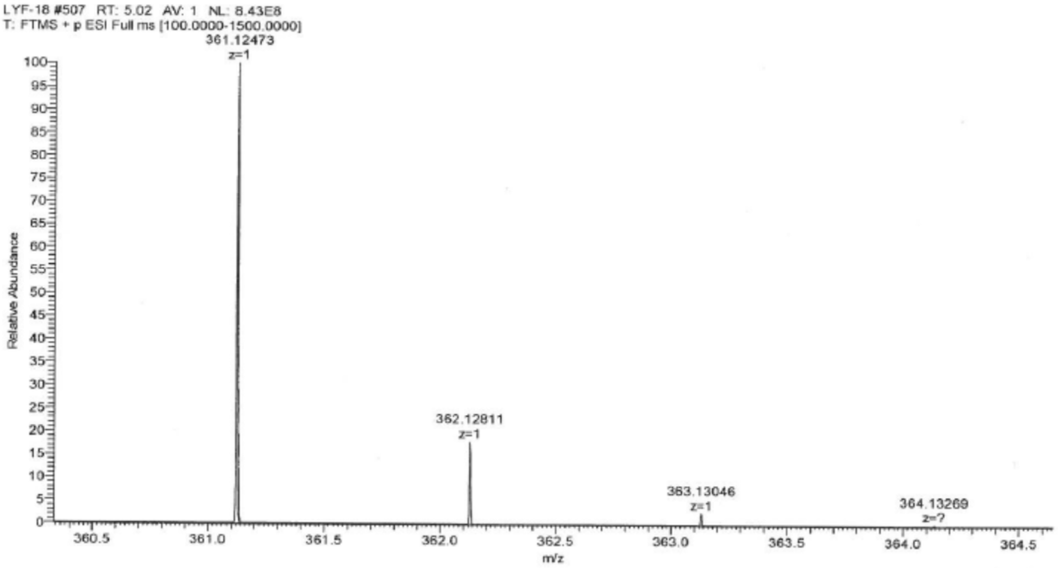

m/z	Theo. Mass	Delta (ppm)	RDB equiv.	Composition	
361.12473	361.12577	-2.89	6.5	C17 H22 O7 Na	M+Na

S7 HRESIMS spectrum of Compound **1**

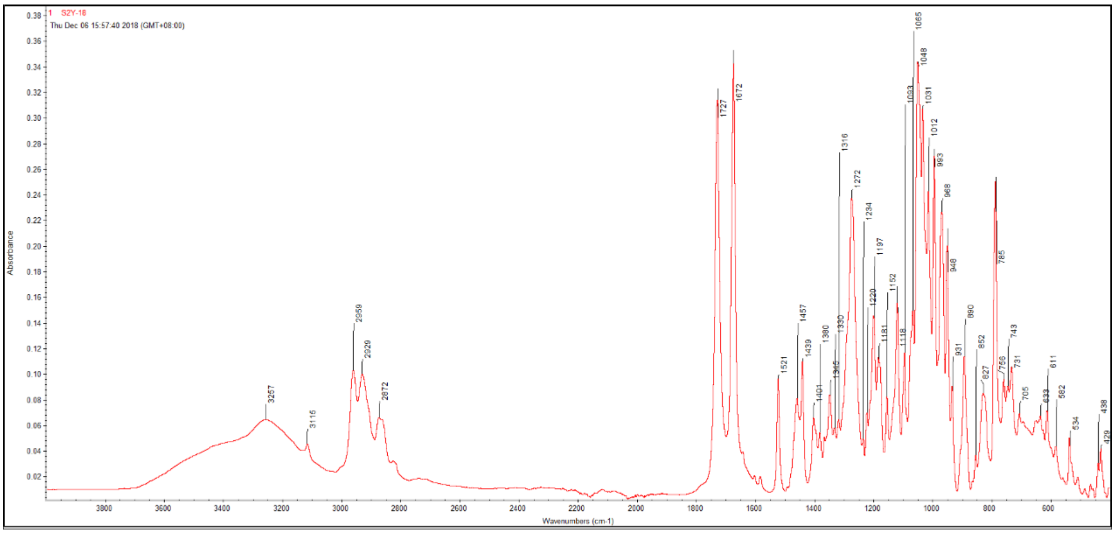

S8 IR spectrum of Compound **1**

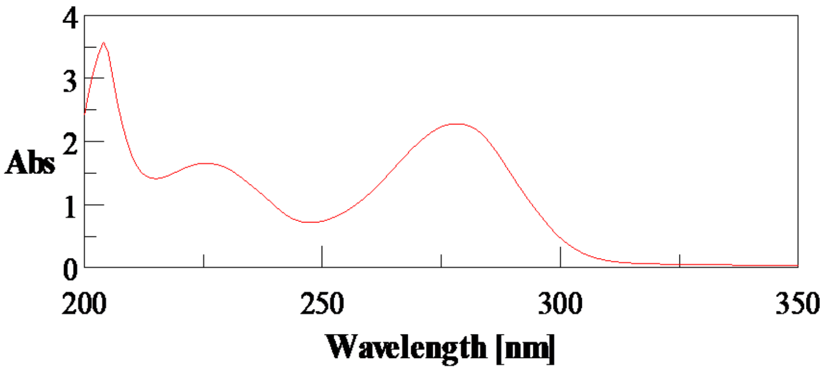

S9 UV spectrum of Compound **1**

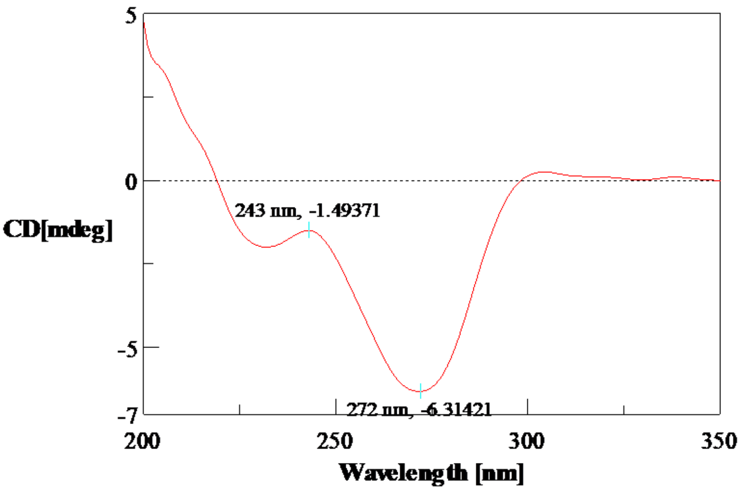

S10 CD spectrum of Compound **1**

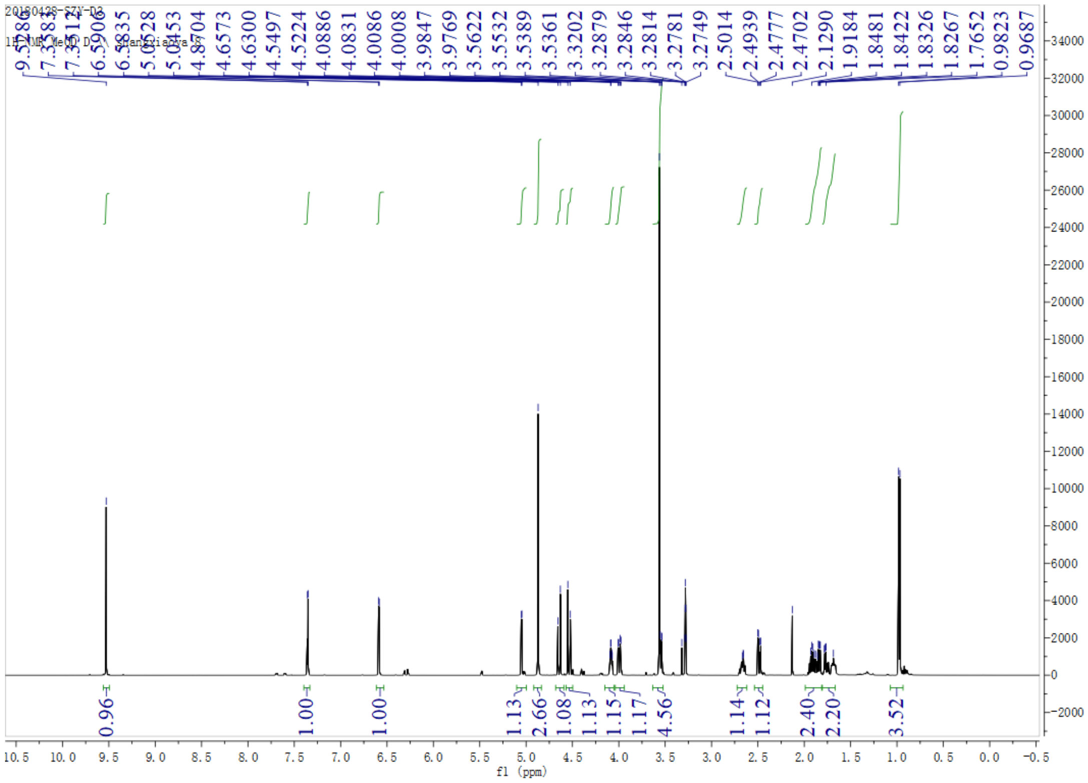

S11 ^{1}H NMR spectrum of Compound **2**

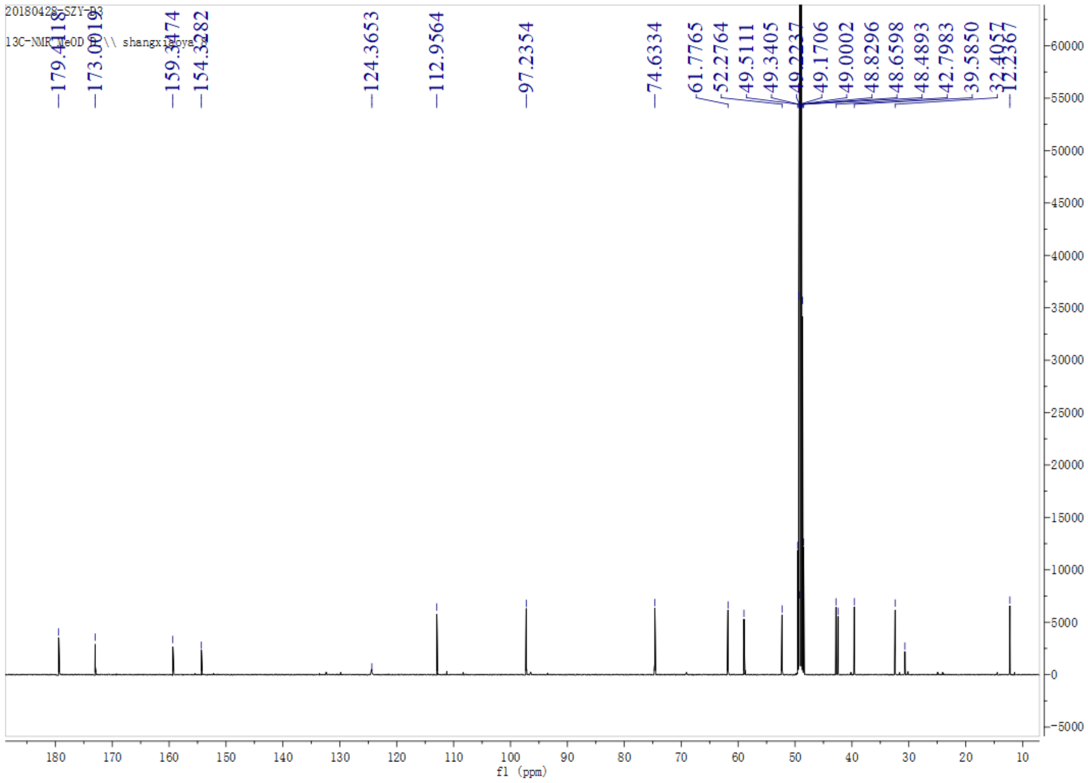

S12 ^{13}C NMR spectrum of Compound **2**

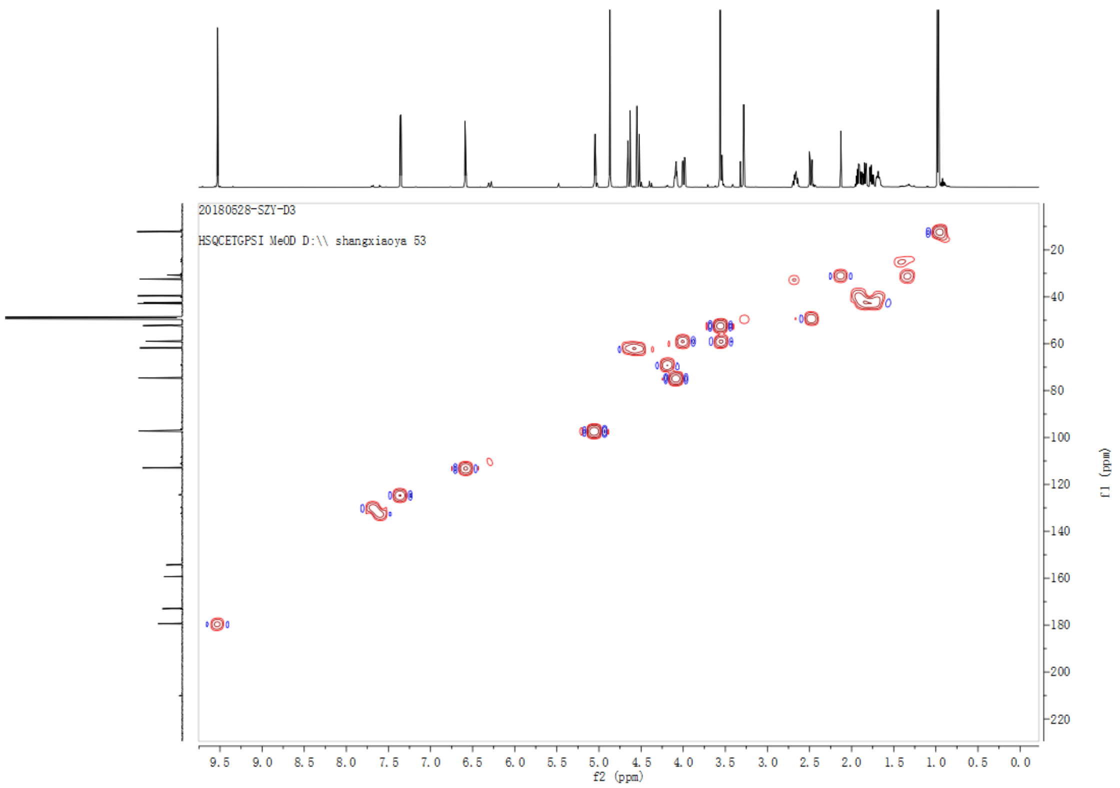

S13 HSQC spectrum of Compound **2**

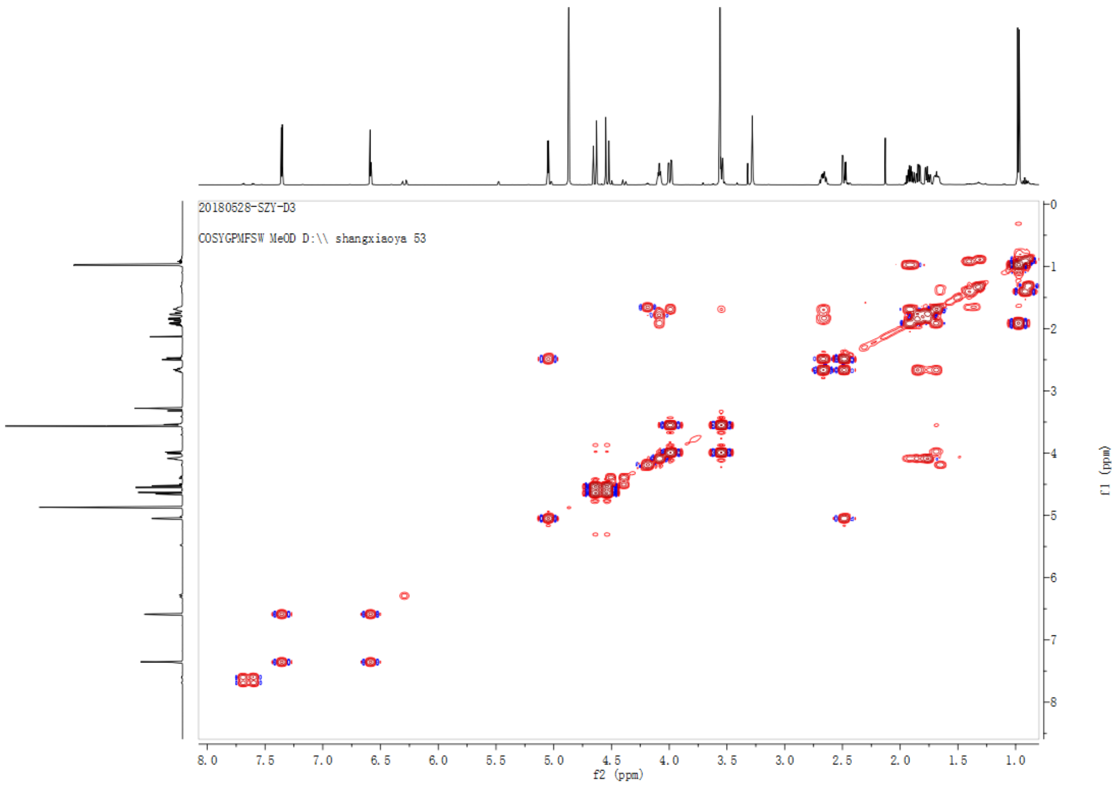

S14 ^{1}H-^{1}H COSY spectrum of Compound **2**

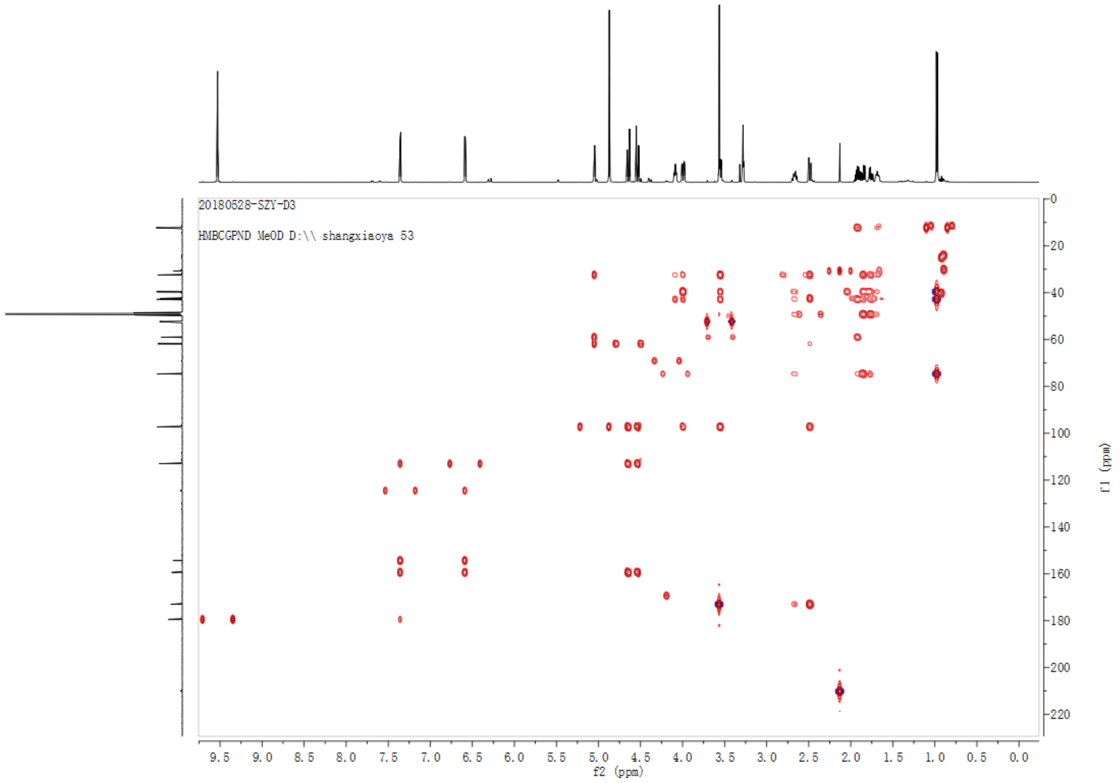

S15 HMBC spectrum of Compound **2**

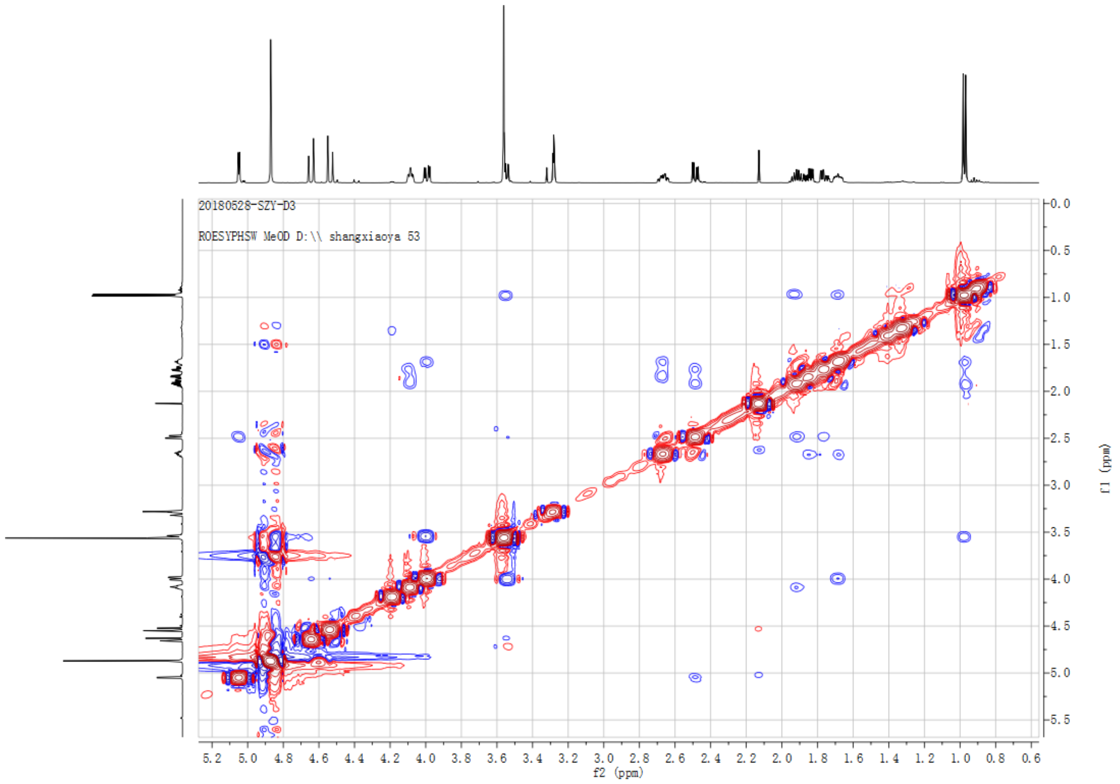

S16 NOESY spectrum of Compound **2**

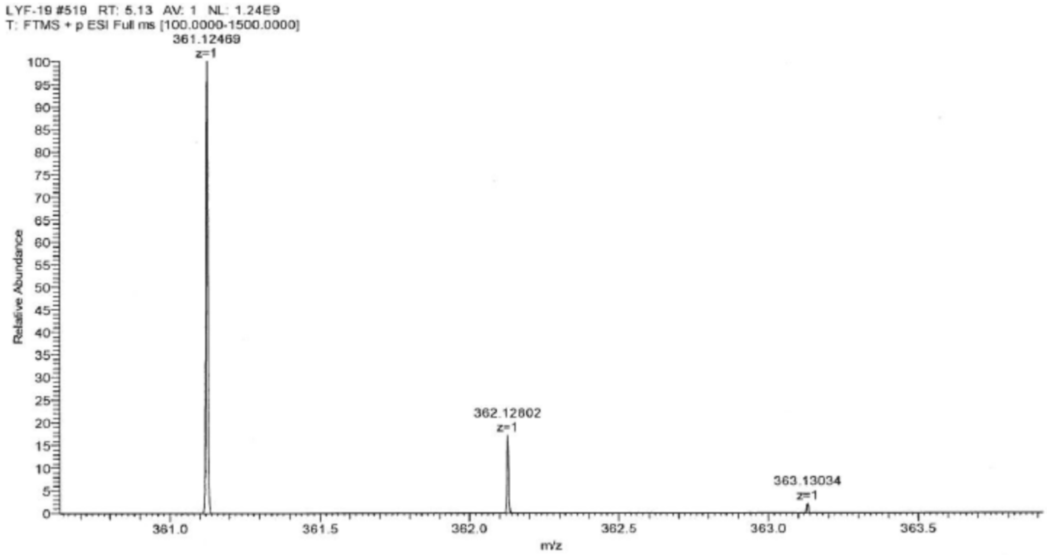

m/z	Theo. Mass	Delta (ppm)	RDB equiv.	Composition	
361.12469	361.12577	-3	6.5	C17 H22 O7 Na	M+Na

S17 HRESIMS spectrum of Compound **2**

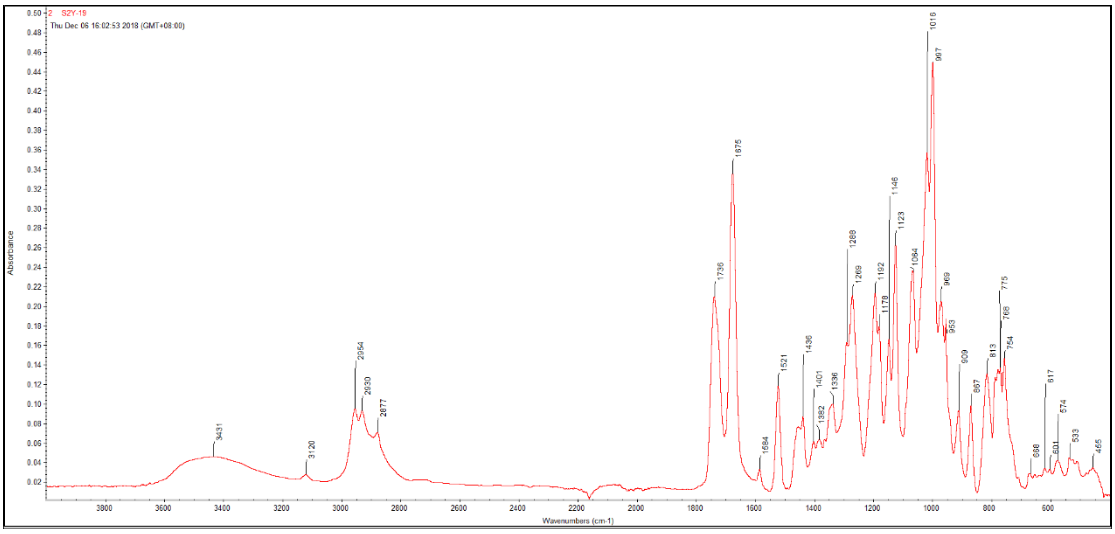

S18 IR spectrum of Compound **2**

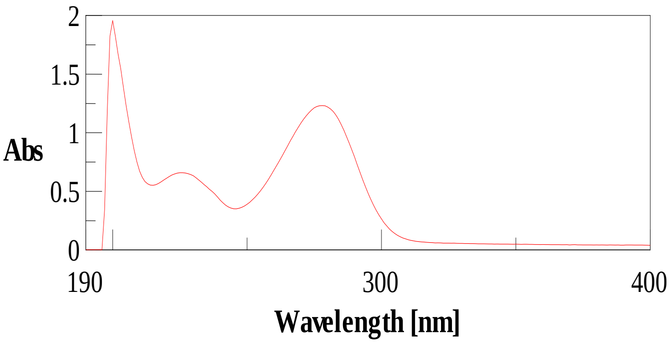

S19 UV spectrum of Compound **2**

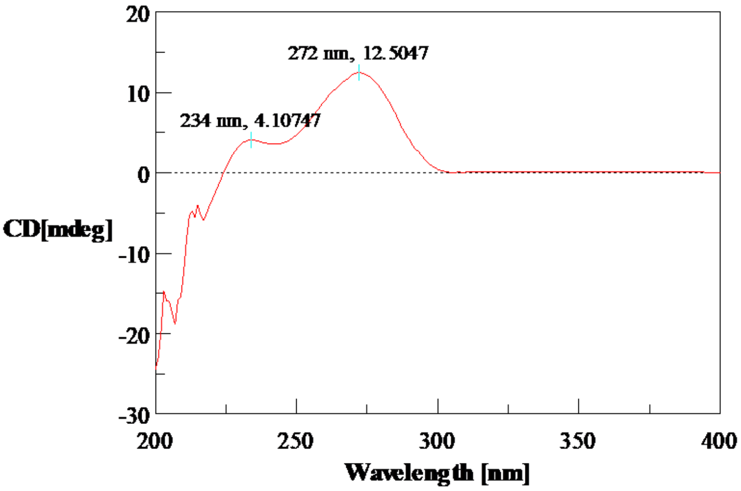

S20 CD spectrum of Compound **2**

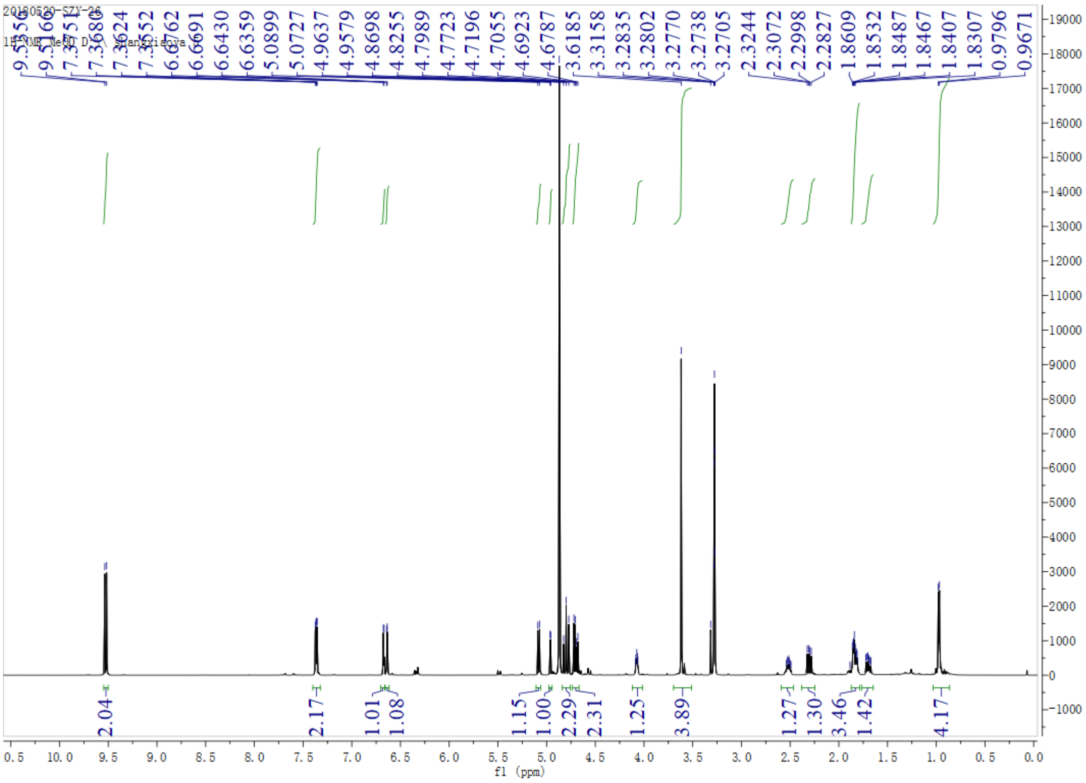

S21 ^{1}H NMR spectrum of Compound **3**

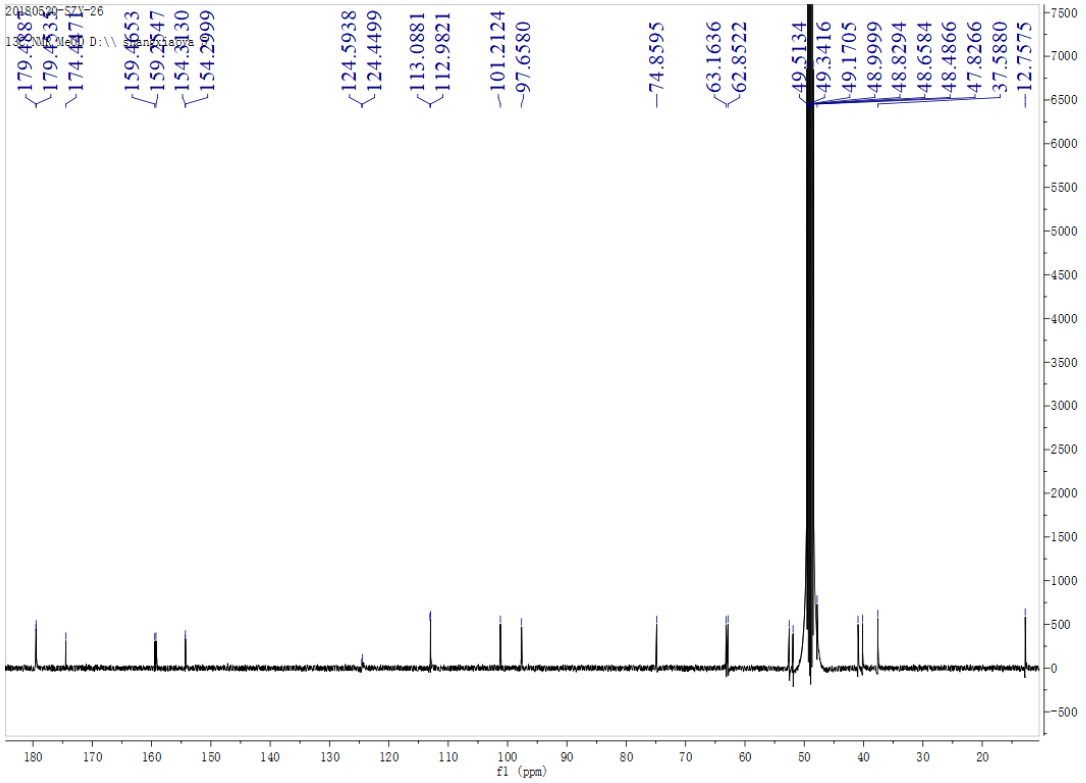

S22 ^{13}C NMR spectrum of Compound **3**

S23 HSQC spectrum of Compound **3**

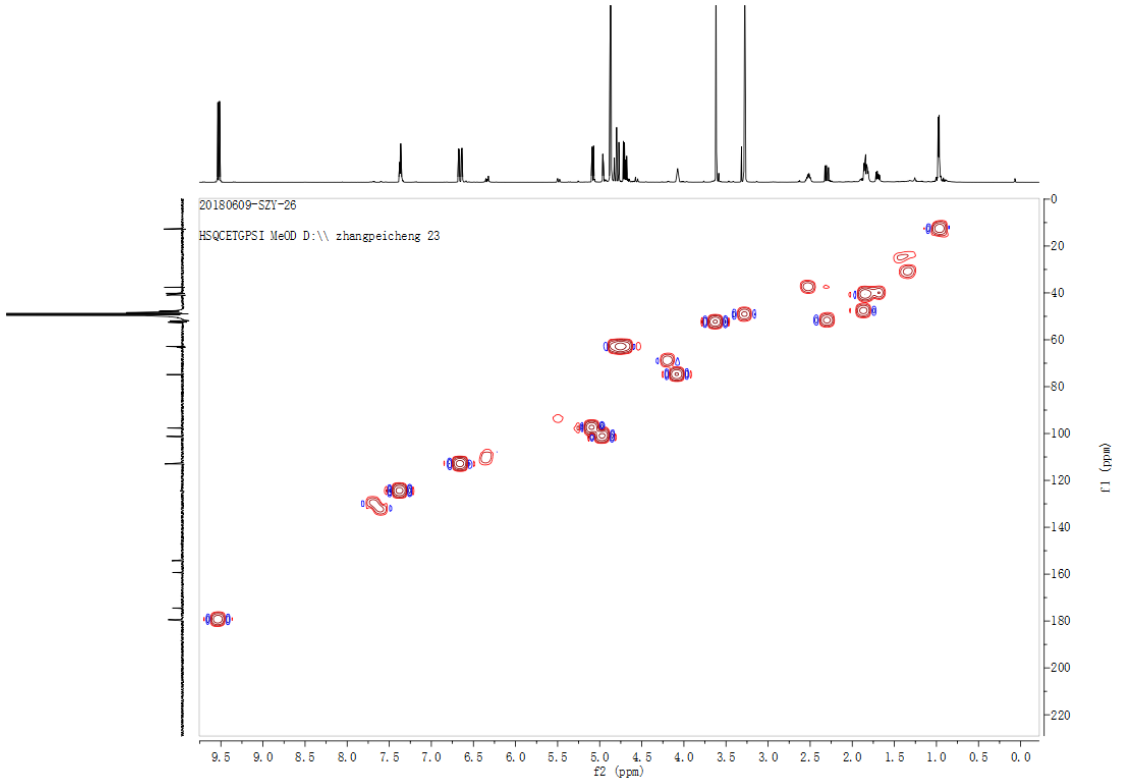

S24 ^{1}H-^{1}H COSY spectrum of Compound **3**

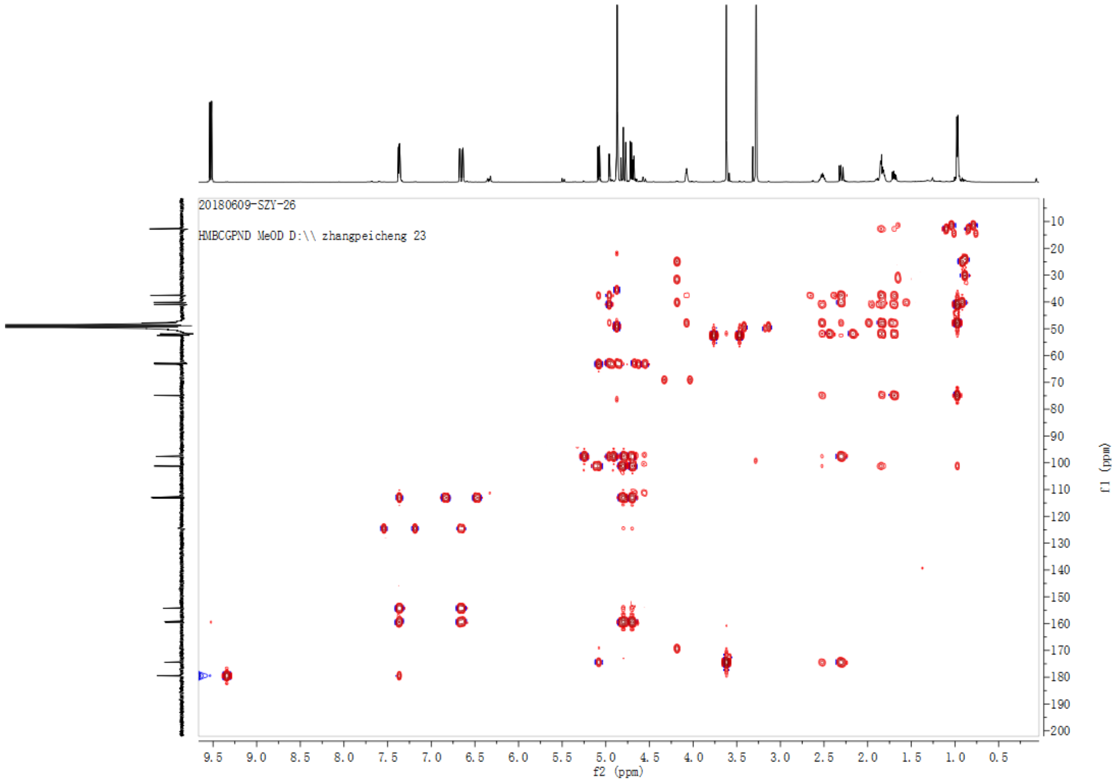

S25 HMBC spectrum of Compound **3**

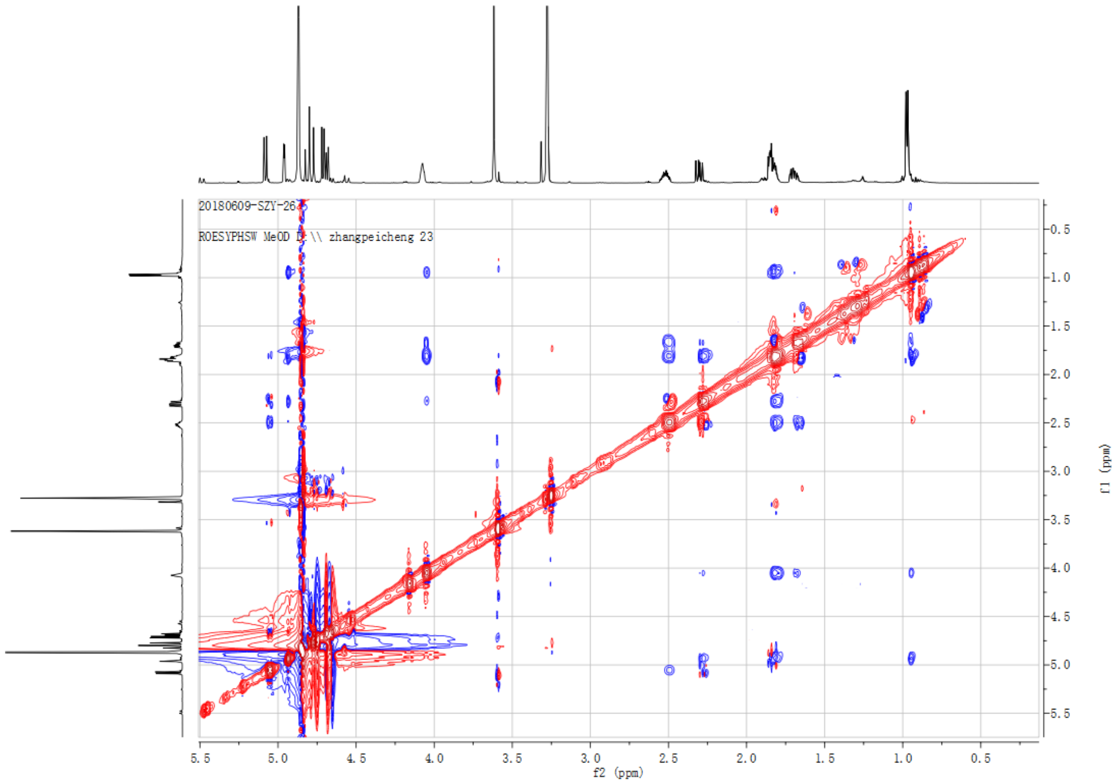

S26 NOESY spectrum of Compound **3**

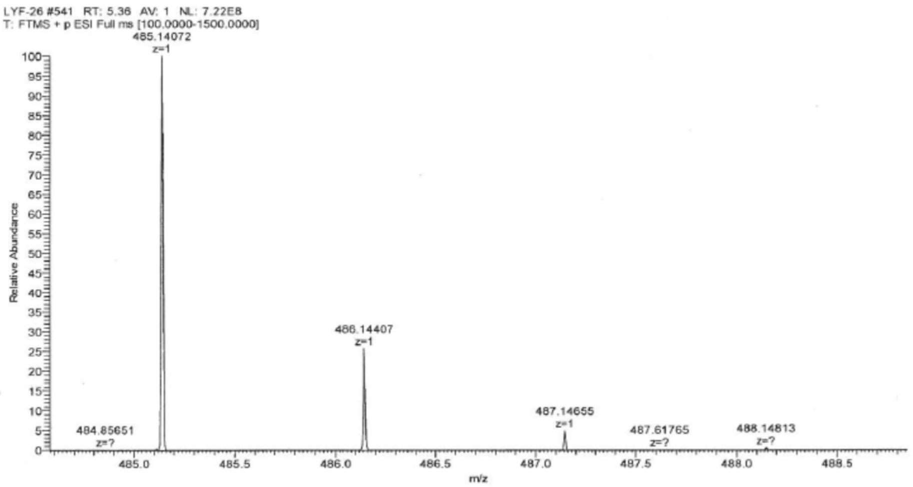

m/z	Theo. Mass	Delta (ppm)	RDB equiv.	Composition	
485.14072	485.14182	-2.26	10.5	C23 H26 O10 Na	M+Na

S27 HRESIMS spectrum of Compound **3**

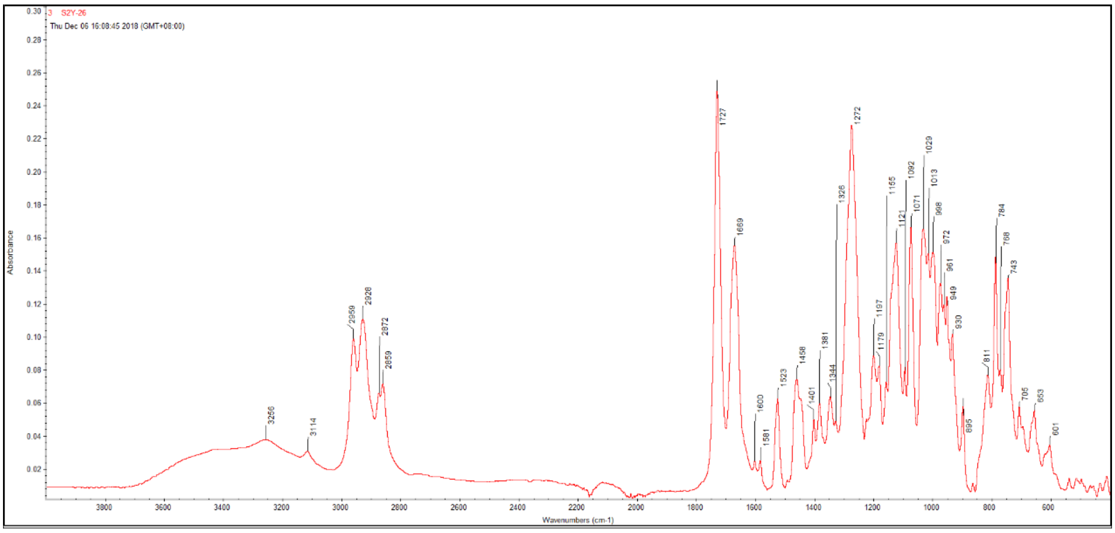

S28 IR spectrum of Compound **3**

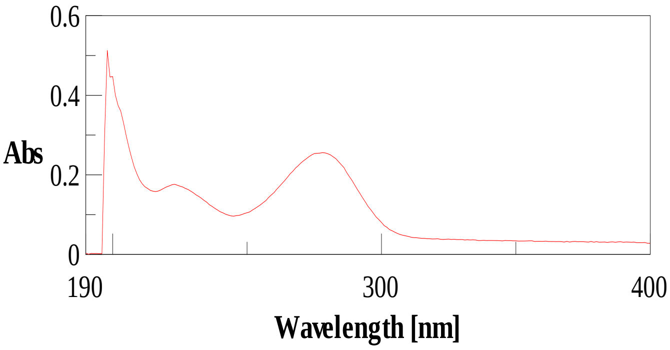

S29 UV spectrum of Compound **3**

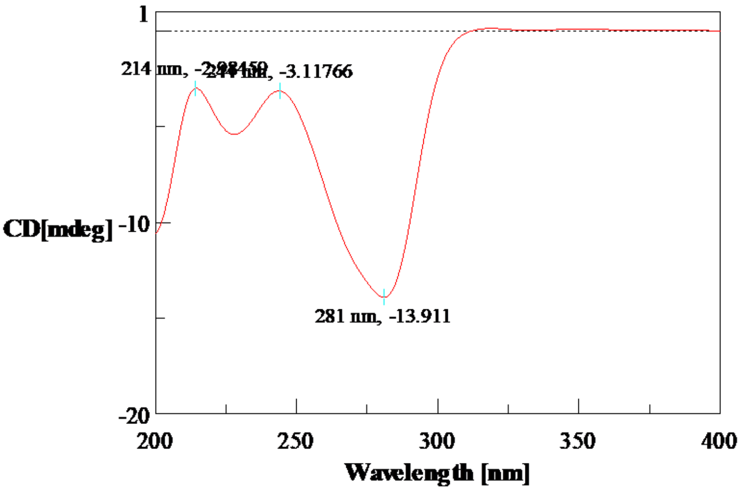

S30 CD spectrum of Compound **3**
